# Developing an Explainable Prognostic Model for Acute Ischemic Stroke: Combining Clinical and Inflammatory Biomarkers With Machine Learning

**DOI:** 10.1002/brb3.70673

**Published:** 2025-07-31

**Authors:** Linlin Ma, Lang Ji, Zhe Cheng, Xiaokun Geng, Yuchuan Ding

**Affiliations:** ^1^ Department of Neurology Beijing Luhe Hospital Capital Medical University Beijing China; ^2^ Central Laboratory Beijing Luhe Hospital Capital Medical University Beijing China; ^3^ China‐America Institute of Neuroscience Beijing Luhe Hospital, Capital Medical University Beijing China; ^4^ Department of Neurosurgery Wayne State University School of Medicine Detroit Michigan USA

**Keywords:** biological indicators, machine learning, prognosis, SHAP

## Abstract

**Background:**

Predicting the prognosis of patients with acute cerebral infarction (ACI) is crucial for clinical decision‐making and personalized treatment. However, existing models often lack the comprehensive integration of clinical and biological indicators necessary for accurate and interpretable predictions. This study aims to develop and validate a predictive model using a combination of clinical assessments and inflammatory biomarkers to improve the prognostication of ACI patients.

**Methods:**

This real‐world, retrospective cohort study was conducted at Luhe Hospital, Beijing, and included 1,017 ACI patients admitted within 24 h of symptom onset. The dataset was randomly split into a training set (80%) and a validation set (20%). Twelve machine learning models were developed and evaluated, with the optimal model and feature set selected based on comprehensive performance metrics. To enhance interpretability, the Shapley Additive exPlanations (SHAP) method was employed to quantify and visualize the contribution of each feature to the model's predictions.

**Results:**

The final model, utilizing the Logistic Regression (LR) algorithm, incorporated six key features: NIHSS at 24 h (NIHSS_24 h), NIHSS_change, D‐dimer, neutrophil count (N), lymphocyte percentage at 24 h (L_pct_24 h), and length of stay (LOS). NIHSS_24 h emerged as a critical early prognostic indicator, effectively predicting three‐month outcomes post‐discharge. Inflammatory markers, including D‐dimer, N, and L_pct_24 h, significantly enhanced the model's predictive performance. The SHAP method provided both global and local interpretability, elucidating the relative importance of each feature in the model's predictions. To facilitate clinical decision‐making, a web‐based application was developed for real‐time prognostic assessment.

**Conclusion:**

This study developed a robust and interpretable predictive model for ACI prognosis by integrating clinical and inflammatory biomarkers. The model underscores the prognostic significance of NIHSS_24 h and inflammatory markers, highlighting the critical role of early assessment and personalized treatment strategies. Future research should focus on multi‐center validation and the incorporation of additional predictive variables to further enhance the model's accuracy and generalizability.

## Graphical abstract

We developed a clinically interpretable ML model to predict 90‐day functional outcomes in acute ischemic stroke using early NIHSS scores and inflammatory markers. Now available as a real‐time web tool to support individualized decision‐making. https://predict‐aci.streamlit.app #StrokeCare #NeuroAI #SHAP #ClinicalAI #TranslationalMedicine

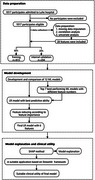

## 1 Introduction

Stroke remains the second leading cause of mortality and disability worldwide, disproportionately affecting low‐ and middle‐income countries (Saini et al. 2021). Acute ischemic stroke results from a sudden cessation of cerebral blood flow, usually due to embolism or thrombosis, causing neuronal damage and subsequent functional impairments (Feigin et al. 2017; Benjamin et al. 2019). Early and accurate prediction of prognosis in ACI patients is crucial for optimizing treatment strategies, resource allocation, and patient counseling (Tong et al. 2023; Lees et al. 2010). The ability to predict outcomes such as survival, functional recovery, and recurrence risk is essential for improving patient care and reducing the burden on healthcare systems (Donnan et al. 2008; Heuschmann et al. 2011; Hankey 2017).

Inflammation is a key component of the pathophysiological response to cerebral ischemia. Following an ischemic event, a complex inflammatory cascade is triggered, involving both the central nervous system and peripheral immune responses (Chamorro et al. 2012; Dirnagl 2012). Research has confirmed that the immune system plays a pivotal role in both the acute and chronic phases of ischemic injury, as well as in the long‐term outcomes of stroke. The activation of innate and adaptive immunity, both within the brain and systemically, significantly impacts stroke prognosis and recovery (Iadecola et al. 2020). Elevated levels of inflammatory biomarkers such as C‐reactive protein (CRP) and D‐dimer have been associated with increased stroke severity and worse outcomes (Whiteley et al. 2008). These markers reflect the extent of tissue damage and the body's response to injury, making them valuable predictors of prognosis (Montaner et al. 2008; Vila et al. 2000). Understanding the role of inflammation in ACI can inform the development of targeted therapies and enhance prognostic models (Emsley and Tyrrell 2002).

Machine learning (ML) offers advanced analytical capabilities for handling complex and high‐dimensional datasets, making it a powerful tool for medical prognosis (Obermeyer and Emanuel 2016). In stroke research, ML algorithms can integrate various clinical and biological variables to create predictive models with superior accuracy compared to traditional statistical methods (Chakraborty et al. 2024; Chen et al. 2024). However, although the ML approach is powerful due to the complexity of the model, it is still limited by the difficulty of stating a direct interpretation, as a so‐called “black box” (Azodi et al. 2020; Molnar 2020).

The SHapley Additive exPlanations (SHAP) method provides a framework for interpreting these models by quantifying the contribution of each feature to the prediction (Lundberg et al. 2020). SHAP values enhance the transparency and interpretability of ML models, which is critical for their application in clinical settings where understanding the decision‐making process is as important as the predictions themselves.

The aim of our study is to develop and validate a predictive model for the prognosis of acute cerebral infarction using inflammatory indicators, and some clinical indicators leveraging machine learning techniques. We intend to evaluate the model's performance in terms of discrimination and calibration, ensuring it accurately predicts patient outcomes. Furthermore, we aim to employ the SHAP method to interpret the importance of individual predictors, providing insights into their roles in prognosis. Our ultimate goal is to enhance the understanding of the prognostic value of inflammatory markers, biological features, and other predictors in ACI and to develop a robust predictive tool that can improve clinical decision‐making and patient management, potentially leading to better patient outcomes and more efficient healthcare delivery.

## 2 Methods

### 2.1 Study Population and Data Collection

This real‐world, retrospective cohort study was conducted at Beijing Luhe Hospital, encompassing the period from January 1, 2022, to December 31, 2023. A total of 1,017 patients aged 18 years or older, who had ACI confirmed by computed tomography (CT) or magnetic resonance imaging (MRI) of the brain within 24 h of symptom onset, were retrieved from the hospital's medical records. We collected demographic characteristics, laboratory data (mainly inflammatory indicators), and vital sign measurements, all obtained from the EMR system. In addition, features with more than 10% missing values were excluded from the following analyses to minimize bias resulting from missing data. Missing data (less than 10%) were handled using multiple imputation by chained equations (MICE) to maintain the integrity of the dataset.

### 2.2 Definition of ACI and Its Prognosis

ACI was defined based on clinical symptoms and confirmed by CT or MRI within 48 h of symptom onset. Moreover, the mRS, a simplified scoring system derived from the modified Rankin Scale (mRS), was designed specifically for this study as an indicator of prognosis at 90 days post‐discharge. The mRS scores were assessed via structured telephone follow‐up at 90 days by trained clinicians, with verification from clinic records when available. It assigns scores based on the level of disability or dependency in daily activities, ranging from 0 (no symptoms) to 6 (death). In our study, we adapted the mRS into a binary outcome measure by categorizing scores as follows: MRS < 3: indicates a favorable prognosis, suggesting minimal to moderate symptoms with some level of independence in daily activities. mRS ≥ 3: indicates an unfavorable prognosis, reflecting severe disability or dependency in daily activities. This simplified scale was chosen for its ease of use and ability to provide a clear dichotomy between favorable and unfavorable outcomes in our research population.

### 2.3 Model Development, Comparison, and Validation

The dataset was randomly divided into a training set (80%) and a validation set (20%). Twelve machine learning (ML) models were developed, including adaptive boosting (AdaBoost), artificial neural network (ANN), decision tree (DT), extra trees (ET), gradient boosting machine (GBM), k‐nearest neighbors (KNN), light gradient boosting machine (LightGBM), logistic regression (LR), Naive Bayes, random forest (RF), support vector machine (SVM), and extreme gradient boosting (XGBoost). These models were trained using a comprehensive set of features derived from the selected variables. Model performance was evaluated using standard metrics, including the area under the receiver operating characteristic (ROC) curve (AUC), accuracy, sensitivity, and specificity. Calibration curves were generated to assess the agreement between predicted probabilities and observed outcomes.

To ensure robustness and generalizability, a 10‐fold cross‐validation approach was employed. This method minimizes the risk of overfitting and evaluates the model's consistency across different data subsets. Performance metrics, particularly AUC, were averaged across all 10 iterations to provide a comprehensive assessment of the model's predictive capability.

### 2.4 Feature Selection

Feature selection was performed using a combination of statistical criteria, clinical expertise, and interpretable machine learning techniques. Variables with a pairwise correlation coefficient ≥0.7 were excluded to mitigate multicollinearity, and univariate analysis retained features with a p‐value <0.1 for their association with the primary outcome (90‐day mRS). Additionally, the SHAP method was employed to rank feature importance and provide interpretable explanations for the model's predictions. SHAP values quantified the contribution of each feature, highlighting the influence of inflammatory indicators and other clinical variables on ACI prognosis. This integrated approach ensured a robust, interpretable, and clinically relevant set of predictors for the model.

### 2.5 Web Application Development Tool Based on Streamlit Framework

A web‐based application was developed using the Streamlit framework, a Python‐based tool designed for creating interactive and user‐friendly web applications. This application enables clinicians to interactively explore and visualize the results of the prediction model. It features an intuitive interface that allows users to input patient data and obtain real‐time prognostic predictions, facilitating clinical decision‐making and personalized patient care.

### 2.6 Statistical Analysis

Continuous variables were expressed as means ± standard deviations and compared using Student's t‐test or the Mann‐Whitney U test, depending on the data distribution. Categorical variables were expressed as counts and percentages and compared using the chi‐square test or Fisher's exact test, as appropriate. The AUCs were used to evaluate the predictive power. A two‐tailed P value < 0.05 was considered statistically significant. All statistical analyses were performed using Python (version 3.12.5).

## 3 Result

### 3.1 Patient Characteristics

**FIGURE 1:**
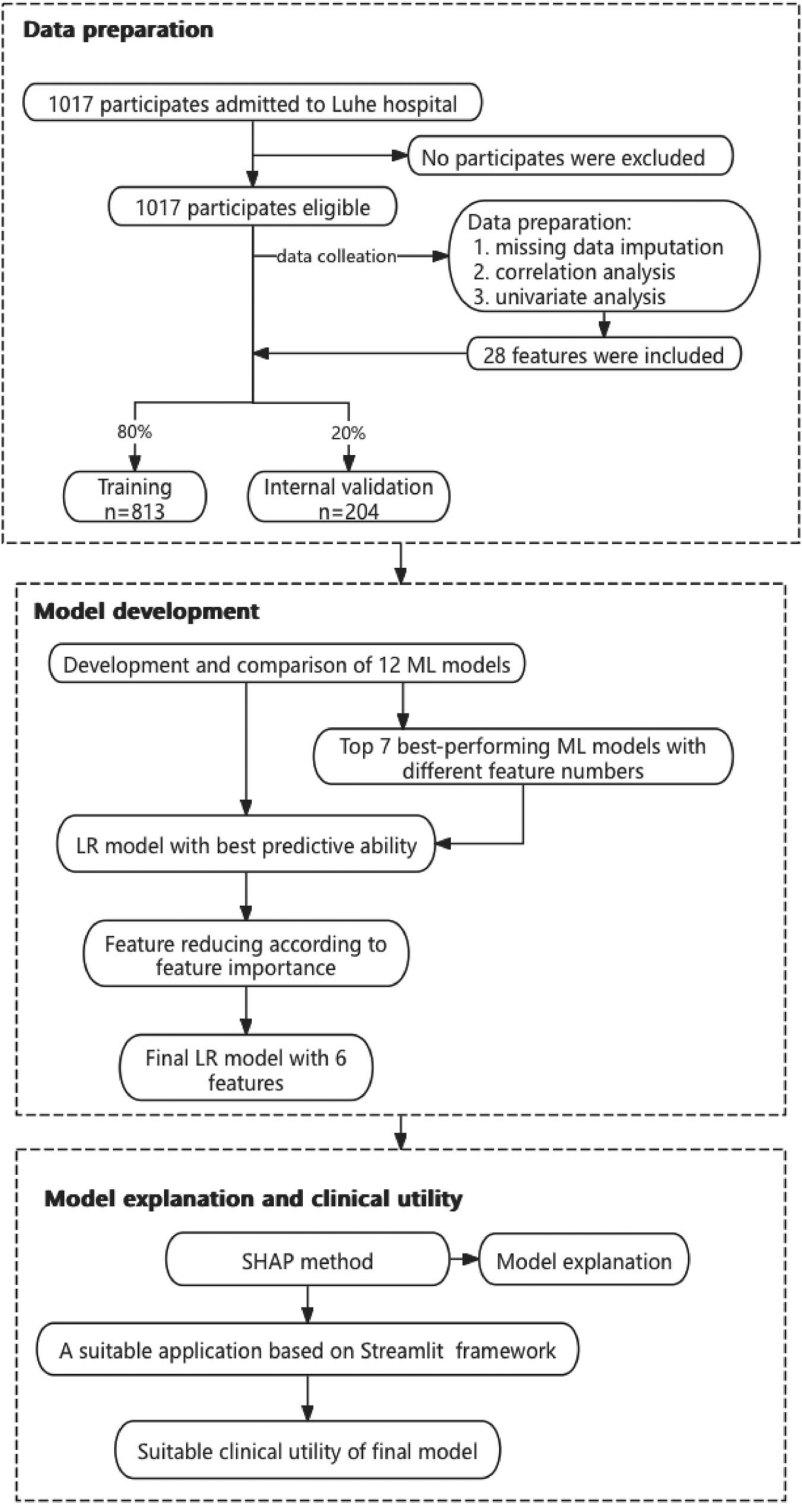
Flow diagram of the study design. LR: logistic regression; ML: machine learning; SHAP: SHapley Additive exPlanation.

In this real‐world, retrospective cohort study, we identified 1,017 patients with ACI from medical records within 24 h of symptom onset for the development of a predictive model. The cohort was divided into a training set (n = 813, 80%) and an internal validation set (n = 204, 20%). The study design is illustrated in Figure 1. Table 1 summarizes the demographic and clinical characteristics of the patients, stratified by prognosis into favorable and unfavorable outcome groups.

**TABLE 1:** Demographic and clinical characteristics associated with favorable and unfavorable prognosis in this real‐world study. Abbreviations: LOS, Length of Stay; AF, Atrial Fibrillation; WBC, White Blood Cell count; N_pct, Neutrophil Percentage; L_pct, Lymphocyte Percentage; M_pct, Monocyte Percentage; N, Neutrophil count; L, Lymphocyte count; M, Monocyte count; RBC, Red Blood Cell count; HB, Hemoglobin; PLT, Platelet count; NTproBNP, N‐terminal pro‐B‐type Natriuretic Peptide; D‐dimer, D‐dimer; GLU, Glucose; EM_SBP, Emergency Systolic Blood Pressure; EM_DBP, Emergency Diastolic Blood Pressure; INHOS_SBP, In‐hospital Systolic Blood Pressure; INHOS_DBP, In‐hospital Diastolic Blood Pressure; WBC_24 h, White Blood Cell count at 24 h; N_pct_24 h, Neutrophil Percentage at 24 h; L_pct_24 h, Lymphocyte Percentage at 24 h; M_pct_24 h, Monocyte Percentage at 24 h; N_24 h, Neutrophil count at 24 h; L_24 h, Lymphocyte count at 24 h; M_24 h, Monocyte count at 24 h; RBC_24 h, Red Blood Cell count at 24 h; HB_24 h, Hemoglobin at 24 h; PLT_24H, Platelet count at 24 h; D‐dimer_24 h, D‐dimer at 24 h; TG, Triglycerides; OTTP, Onset To Time Of Presentation; Ad_NIHSS, Admission NIH Stroke Scale; NIHSS_24 h, NIH Stroke Scale at 24 h; NIHSS change, Change in NIH Stroke Scale; CTble, CT Bleeding.

		Favorable	Unfavorable	P‐Value
Sex, n (%)				
	Male	424 (71.9)	262 (61.4)	0.001
	Female	166 (28.1)	165 (38.6)	
Age, y; mean (SD)		65.7 (11.6)	69.8 (12.4)	<0.001
Smoking, n (%)				
	No	270 (45.8)	235 (55.0)	0.014
	Yes	320 (54.2)	192 (45.0)	
Drinking, n (%)				
	No	372 (63.1)	294 (68.9)	0.064
	Yes	218 (36.9)	133 (31.1)	
LOS, d; mean (SD)		8.3 (9.7)	15.0 (24.8)	<0.001
AF, n (%)				
	No	514 (87.1)	342 (80.1)	0.003
	Yes	76 (12.9)	85 (19.9)	
WBC, 10^9/L; mean (SD)		8.4 (3.7)	8.9 (3.8)	0.053
N_pct, mean (SD)		70.1 (10.8)	72.5 (12.1)	0.001
L_pct, mean (SD)		23.0 (9.7)	20.8 (10.5)	0.001
M_pct, mean (SD)		5.1 (2.3)	4.9 (2.0)	0.185
N, 10^9/L; mean (SD)		5.5 (2.4)	6.0 (2.8)	0.001
L, 10^9/L; mean (SD)		1.7 (0.8)	1.6 (0.9)	0.149
M, 10^9/L; mean (SD)		0.4 (0.1)	0.4 (0.4)	0.027
RBC, 10^12/L; mean (SD)		4.6 (0.5)	4.5 (0.7)	0.096
HB, g/L; mean (SD)		143.8 (19.1)	141.2 (21.2)	0.045
PLT, 10^9/L; mean (SD)		216.3 (63.9)	211.6 (65.7)	0.256
NTproBNP, pg/ml; mean (SD)		547.9 (1766.4)	926.2 (2434.9)	0.006
D‐dimer, mg/L; mean (SD)		0.5 (0.7)	1.0 (1.7)	<0.001
GLU, mmol/L; mean (SD)		8.7 (3.5)	9.4 (3.9)	0.003
EM_SBP, mmHg; mean (SD)		142.4 (21.6)	148.1 (22.8)	<0.001
EM_DBP, mmHg; mean (SD)		83.0 (12.0)	83.2 (12.6)	0.838
INHOS_SBP, mmHg; mean (SD)		152.1 (23.2)	156.1 (24.0)	0.009
INHOS_DBP, mmHg; mean (SD)		85.4 (14.1)	86.2 (16.0)	0.385
WBC_24 h, 10^9/L; mean (SD)		7.4 (2.2)	9.2 (3.2)	<0.001
N_pct_24 h, mean (SD)		69.3 (9.9)	78.7 (10.2)	<0.001
L_pct_24 h, mean (SD)		21.7 (10.8)	13.7 (8.5)	<0.001
M_pct_24 h, mean (SD)		7.4 (2.2)	6.6 (2.6)	<0.001
N_24 h, 10^9/L; mean (SD)		5.4 (2.5)	7.4 (3.2)	<0.001
L_24 h, 10^9/L; mean (SD)		1.5 (0.6)	1.1 (0.7)	<0.001
M_24 h, 10^9/L; mean (SD)		0.5 (0.3)	0.6 (0.2)	0.021
RBC_24 h, 10^12/L; mean (SD)		4.3 (0.5)	4.2 (0.6)	0.006
HB_24 h, g/L; mean (SD)		134.5 (16.5)	130.9 (20.2)	0.003
PLT_24H, 10^9/L; mean (SD)		202.4 (62.5)	199.8 (67.3)	0.529
D‐dimer_24 h, mg/L; mean (SD)		1.5 (3.5)	2.4 (4.0)	<0.001
TG, mmol/L; mean (SD)		1.5 (3.1)	1.2 (0.8)	0.012
OTTP, min; mean (SD)		320.0 (394.5)	252.7 (297.4)	0.002
Ad_NIHSS, mean (SD)		6.1 (6.2)	13.5 (7.3)	<0.001
NIHSS_24 h, mean (SD)		2.9 (3.3)	13.7 (8.8)	<0.001
NHISS change, mean (SD)		−3.3 (5.1)	0.1 (5.9)	<0.001
CTble, n (%)				
	No	564 (95.6)	339 (79.4)	<0.001
	Yes	26 (4.4)	88 (20.6)	

### 3.2 Feature Selection

**FIGURE 2:**
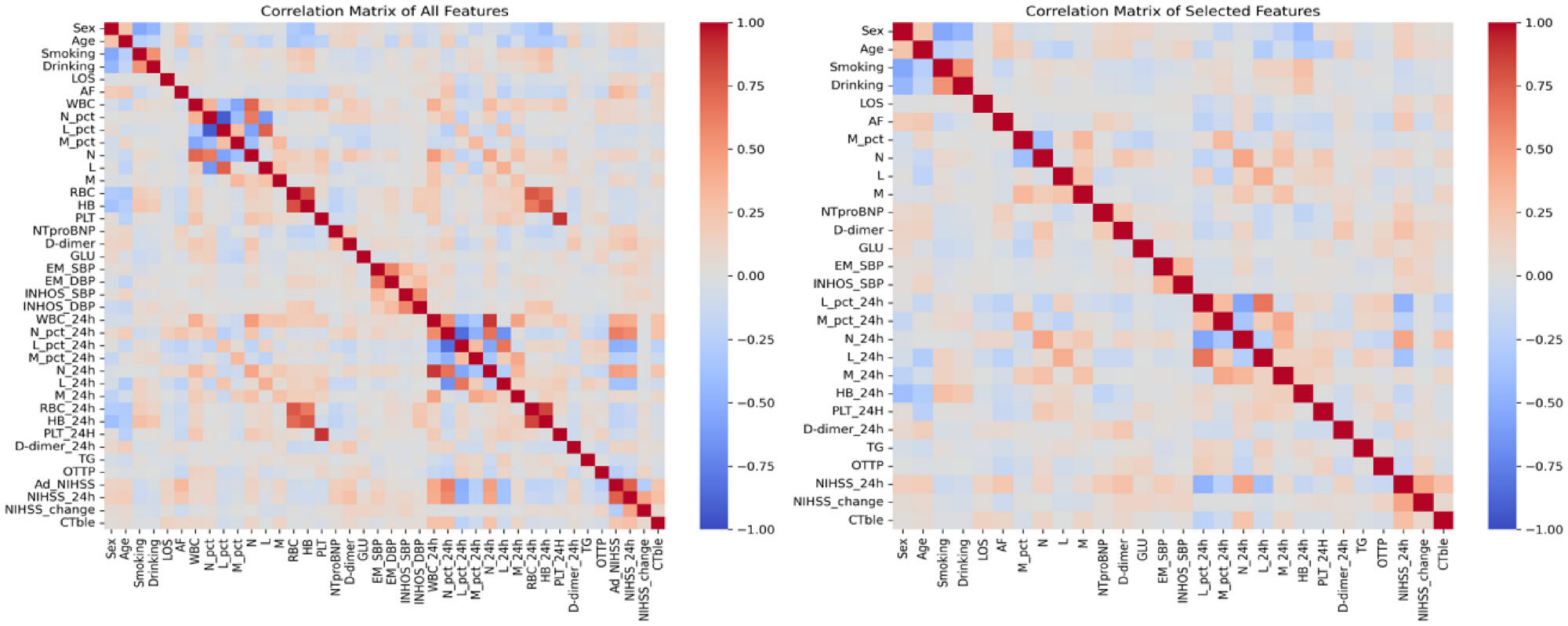
**Correlation Matrix of Features. Left Panel**: Correlation matrix of all features. **Right Panel**: Correlation matrix of selected features.

The correlation matrix (Figure 2) was used to evaluate relationships among all features, with the left panel displaying correlations across all variables and the right panel highlighting features with a correlation coefficient ≤0.7.

Based on the univariate analysis (Table 1) and correlation matrix, a total of 28 features were utilized to develop the prediction models, including Sex, Age, Smoking, Drinking, length of stay (LOS), Atrial Fibrillation (AF), Monocyte Percentage (M_pct), Neutrophil Count (N), Lymphocyte Count (L), Monocyte Count (M), N‐Terminal Pro‐B‐Type Natriuretic Peptide (NTproBNP), D‐dimer, Glucose (GLU), Emergency Systolic Blood Pressure (EM_SBP), In‐Hospital Systolic Blood Pressure (INHOS_SBP), Lymphocyte Percentage At 24 h (L_pct_24 h), Monocyte Percentage At 24 h (M_pct_24 h), Neutrophil Count At 24 h (N_24 h), Lymphocyte Count At 24 h (L_24 h), Monocyte Count At 24 h (M_24 h), Hemoglobin Level At 24 h (HB_24 h), Platelet Count At 24 h (PLT_24H), D‐dimer At 24 h (D‐dimer_24 h), Triglycerides (TG), Onset To Time Of Presentation (OTTP), NIH Stroke Scale At 24 h (NIHSS_24 h), Change In NIH Stroke Scale (NIHSS_change), And Computed Tomography Bleeding Event (CT ble). NIHSS_change was calculated as the difference between NIHSS_24 h and the NIHSS score on admission (i.e., NIHSS_24 h minus NIHSS_baseline). These features were selected to comprehensively capture the clinical and inflammatory factors associated with ACI prognosis.

### 3.3 Model Selection and Validation

**FIGURE 3:**
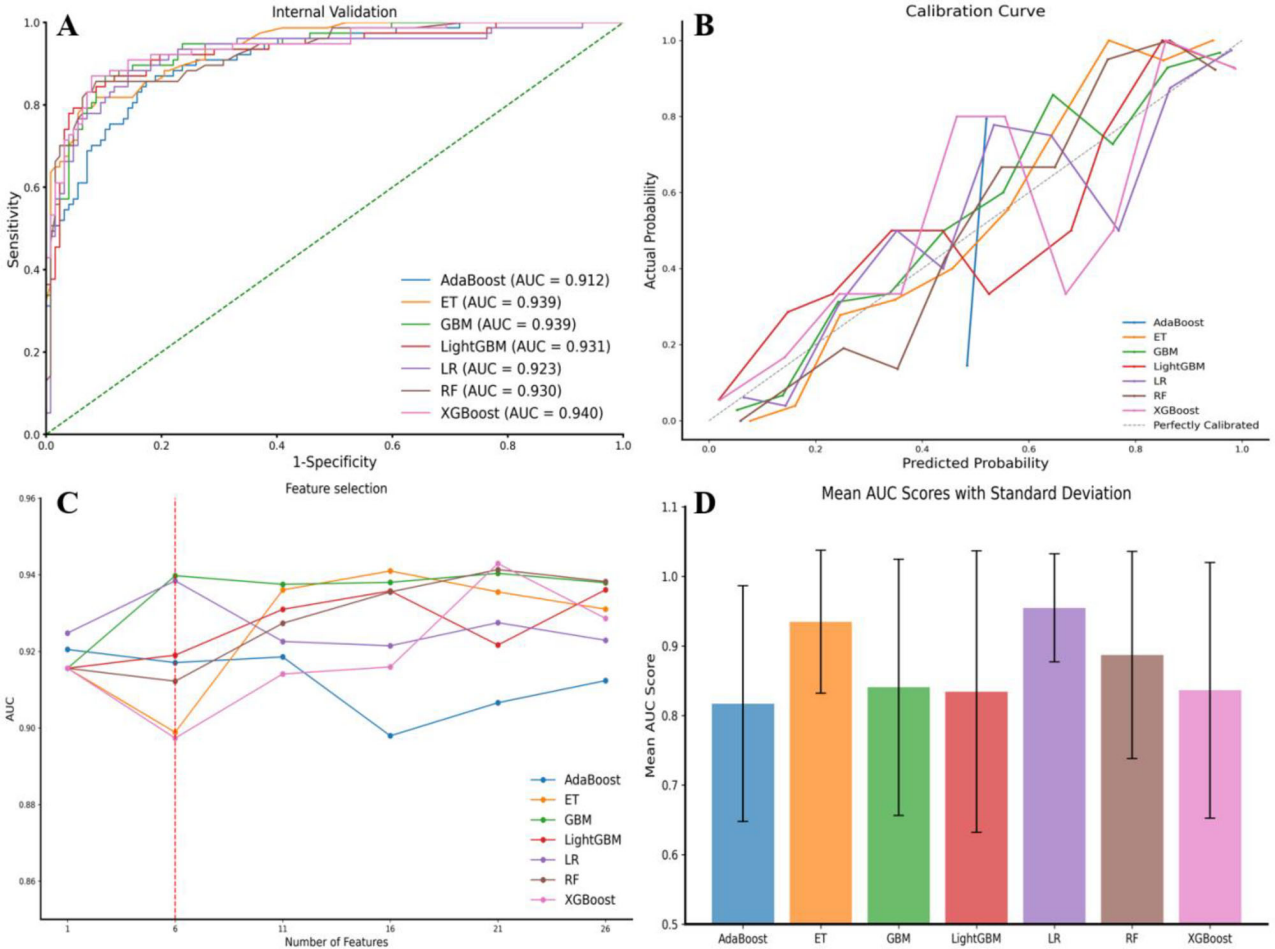
**Performance of ML models in predicting prognosis**. (**A**) ROC curves for the top 7 models. (**B**) Calibration Curve for the top 7 models. (**C**) AUC values for the top 7 models with varying feature counts. (**D**) Ten‐fold cross‐validation for the top 7 models. Abbreviations: AdaBoost, Adaptive Boosting; ET, Extra Trees; GBM, Gradient Boosting Machine; LightGBM, Light Gradient Boosting Machine; LR, Logistic Regression; RF, Random Forest; XGBoost, eXtreme Gradient Boosting; AUC, area under the ROC curve; ROC, receiver operating characteristic.

We developed 12 ML models to predict the prognosis of patients with acute cerebral infarction and subsequently compared their performance, selecting the top 7 models for further evaluation. All selected models demonstrated strong predictive performance, with AUC values as follows: AdaBoost (AUC = 0.912), Extra Trees (ET; AUC = 0.939), Gradient Boosting Machine (GBM; AUC = 0.939), LightGBM (AUC = 0.931), Logistic Regression (LR; AUC = 0.923), Random Forest (RF; AUC = 0.930), and XGBoost (AUC = 0.940), as illustrated in Figure 3A. ROC curves for all 12 models are provided in Appendix S1. Calibration curves were constructed to assess the agreement between predicted probabilities and observed outcomes for the 7 models. All models exhibited good calibration, closely aligning with the ideal calibration curve, except for AdaBoost, which showed slight deviations (Figure 3B).

Feature reduction based on importance rankings revealed that the LR and GBM models maintained superior predictive performance even with fewer features, highlighting their efficiency and interpretability (Figure 3C). The AUC values for the top 7 models, utilizing 1 to 28 features, are detailed in Appendix S2. To evaluate the robustness of the models, we performed 10‐fold cross‐validation. The LR model achieved the highest mean AUC of 0.955 (SD = 0.078), demonstrating excellent consistency across folds. This was followed by the ET model (mean AUC = 0.935, SD = 0.103) and the RF model (mean AUC = 0.887, SD = 0.149). All the ten‐fold cross‐validation results (including mean AUC, standard deviation of AUC, and all scores) for these 7 models are presented in Appendix S3.

Based on these comprehensive evaluations, the LR model, incorporating only 6 key features, was identified as the optimal prognostic model due to its superior predictive accuracy and robustness. Feature selection was guided by SHAP values derived from the LR model, with the SHAP summary plot for all 28 features provided in Appendix S4. The final six features, selected based on their SHAP importance, were: NIHSS_24 h, NIHSS_change, LOS, D‐dimer, L_pct_24 h, and N.

**FIGURE 4:**
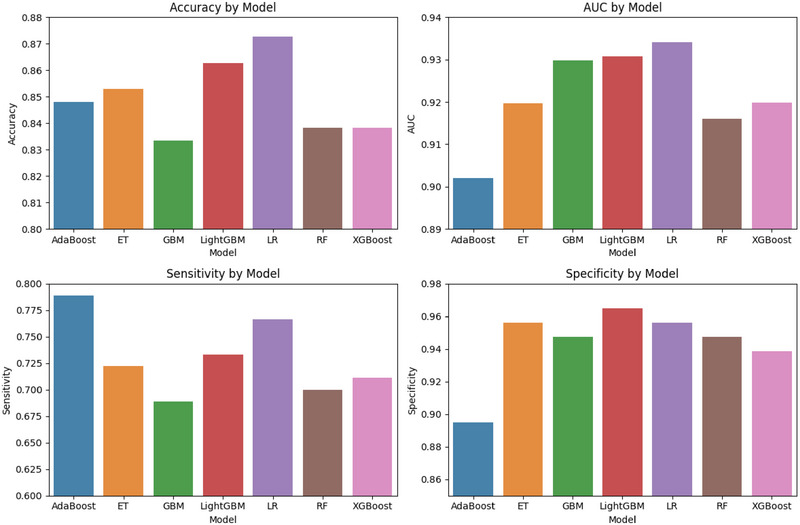
Comparative performance of machine learning models using the selected 6 features.

To further evaluate the performance of the LR model with selected 6 features, we calculated the accuracy, AUC, sensitivity, and specificity for all 7 models. As shown in Figure 4, the LR model demonstrated superior performance across all metrics, particularly in AUC (0.934) and accuracy (0.873), highlighting its robustness and predictive capability. Detailed metrics for the seven models, including accuracy, AUC, sensitivity, and specificity, are provided in Appendix S5.

### 3.4 Model Explanation

**FIGURE 5:**
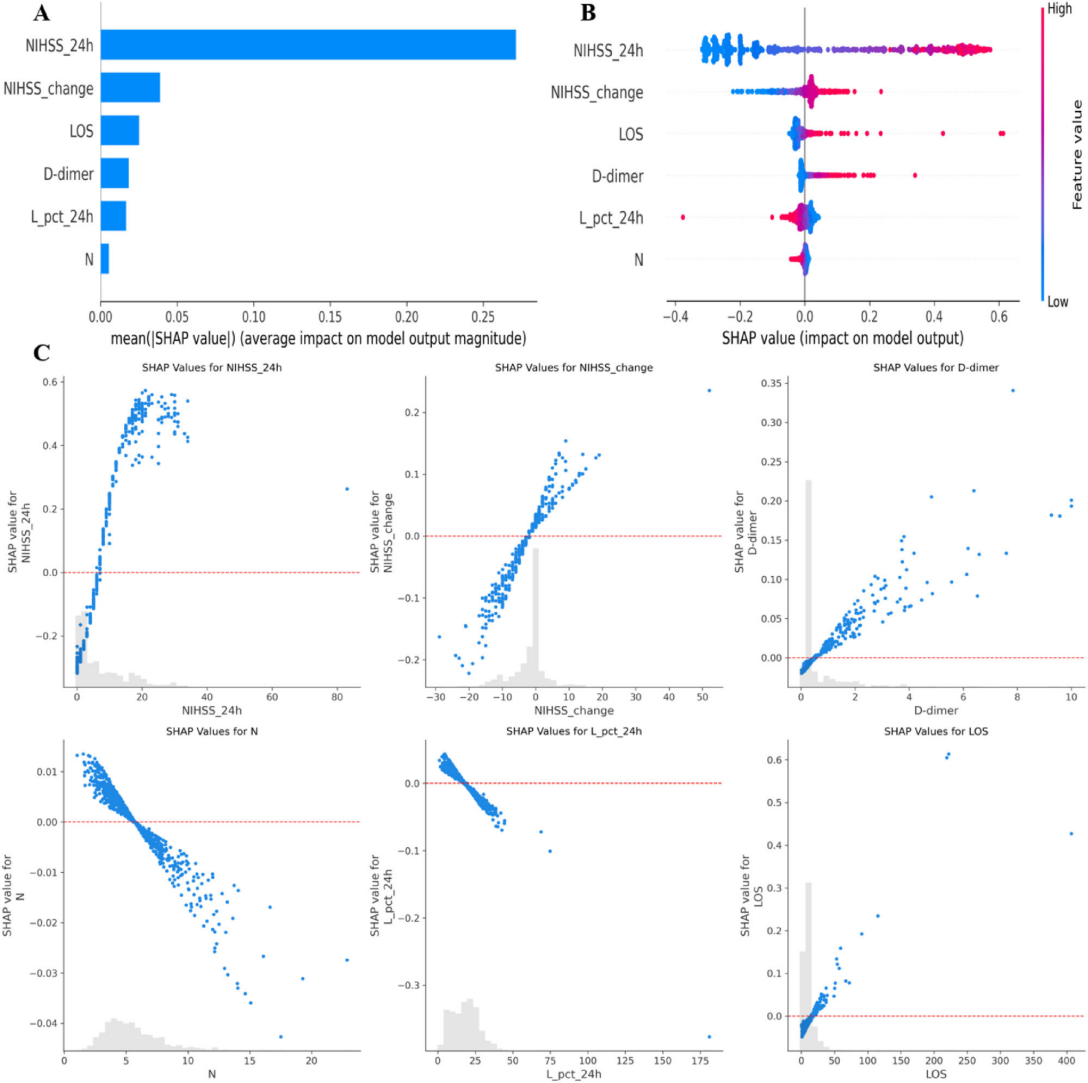
**Global model interpretation via the SHAP method**. (**A**) SHAP summary bar plot. (**B**) SHAP summary dot plot. The probability of prognosis correlates with the SHAP value of a feature. Each dot represents a single patient's SHAP value for a specific feature, with red indicating higher values and blue indicating lower values, stacked vertically to show density. (**C**) SHAP dependence plot. Each plot demonstrates how a single feature impacts the model's output, with each dot representing an individual patient. For example, NIHSS_change ≥ 0 is associated with an unfavorable classification, with SHAP values greater than zero pushing towards “Unfavorable”.

Given the challenge of clinician acceptance for non‐transparent predictive models, we employed the SHAP method to elucidate the contributions of each variable in our final model (Mitchell et al. 2022). This approach offers two types of insights: a global explanation at the feature level and a local explanation at the individual level (Lundberg et al. 2020). The global explanation captures the model's overall functionality, with SHAP summary plots (Figure 5A and B) indicating the importance of each feature based on average SHAP values in descending order. Additionally, SHAP dependence plots clarify how individual features influence the model's output. Figure 5C presents the relationship between real values and SHAP values for the six features, where positive SHAP values correspond to a higher likelihood of unfavorable prognosis. Each point represents an individual patient. The color indicates the value of the feature (red for high, blue for low), and the position along the X‐axis shows whether the feature increases (right) or decreases (left) the predicted risk. For example, higher NIHSS_24 h scores and elevated D‐dimer levels contributed significantly to higher predicted probability of unfavorable outcomes.

**FIGURE 6:**
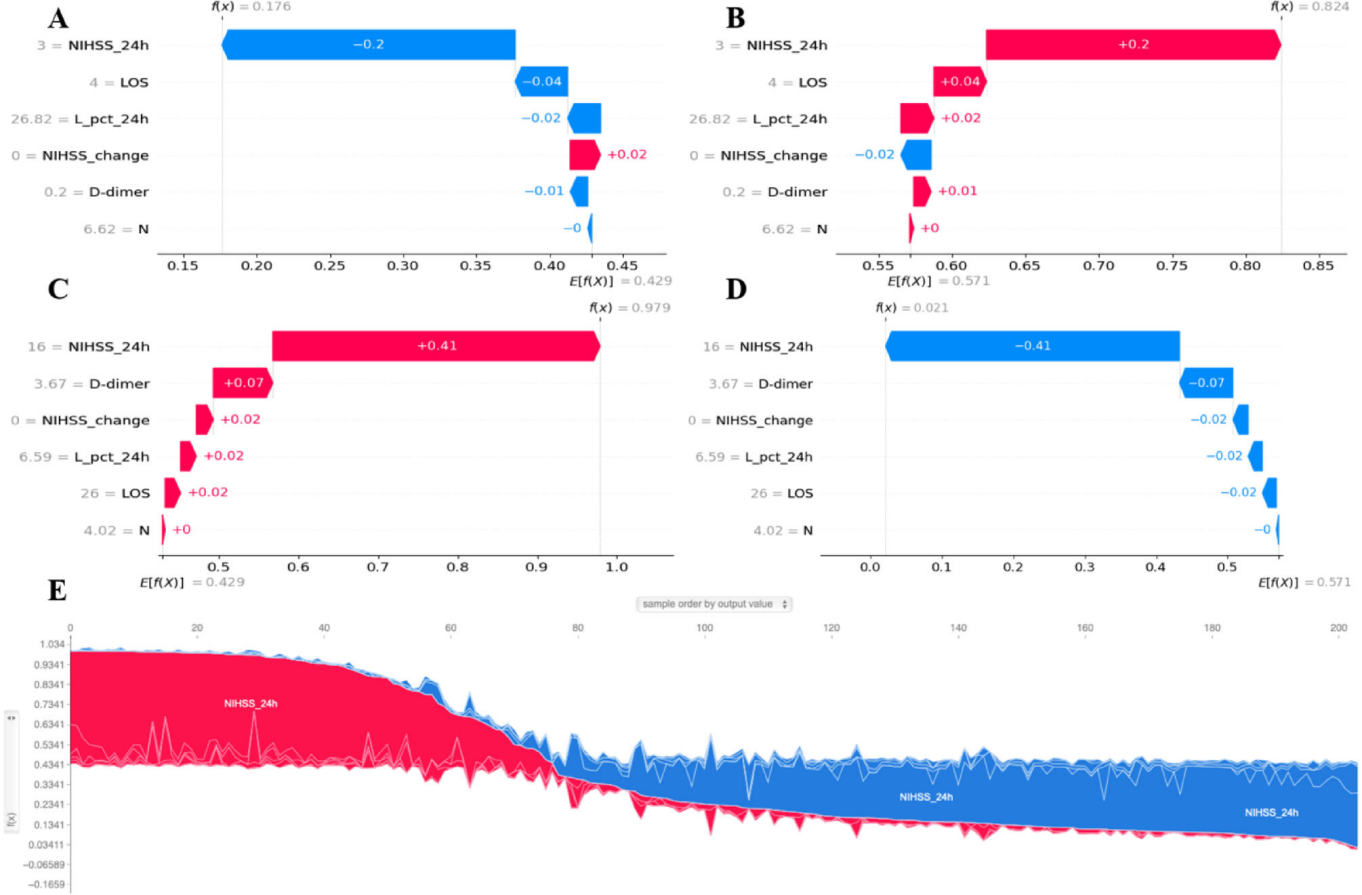
**Local model interpretation using the SHAP method**. (A–E) Waterfall plots illustrate feature contributions for individual patients stratified by low (A–B) or high (C–D) risk of unfavorable outcomes. Panels A and C represent patients whose predicted outcomes trend toward the “Unfavorable” class, while B and D depict patients trending toward the “Favorable” class. (E) Force plot for the internal validation set, with patients on the x‐axis and feature contributions on the y‐axis; increased red shading indicates a higher probability of “Unfavorable” outcomes.

The local explanation further investigates how specific predictions are determined for individual patients by incorporating their unique data inputs. Figures 6A and B illustrate a patient with a favorable prognosis; Figure 6A shows a prediction of “Unfavorable” with a 17.6% probability, while Figure 6B reflects a “Favorable” prediction with an 82.4% probability based on the model. The waterfall plots in these figures display the actual measured values for each feature. Notably, factors such as NIHSS_24 h, LOS, L_pct_24 h, and D‐dimer supported the “Favorable” classification, whereas NIHSS_change did not. A comparable analysis is provided for a patient with an unfavorable prognosis in Figures 6C and D. Additionally, Figure 6E presents a force plot illustrating contributions from features for patients in the internal validation set, with the x‐axis representing each patient and the y‐axis reflecting the contributions of features. An increased red area for each patient indicates a higher probability of an “Unfavorable” classification.

### 3.5 Convenient Application for Clinical Utility

**FIGURE 7:**
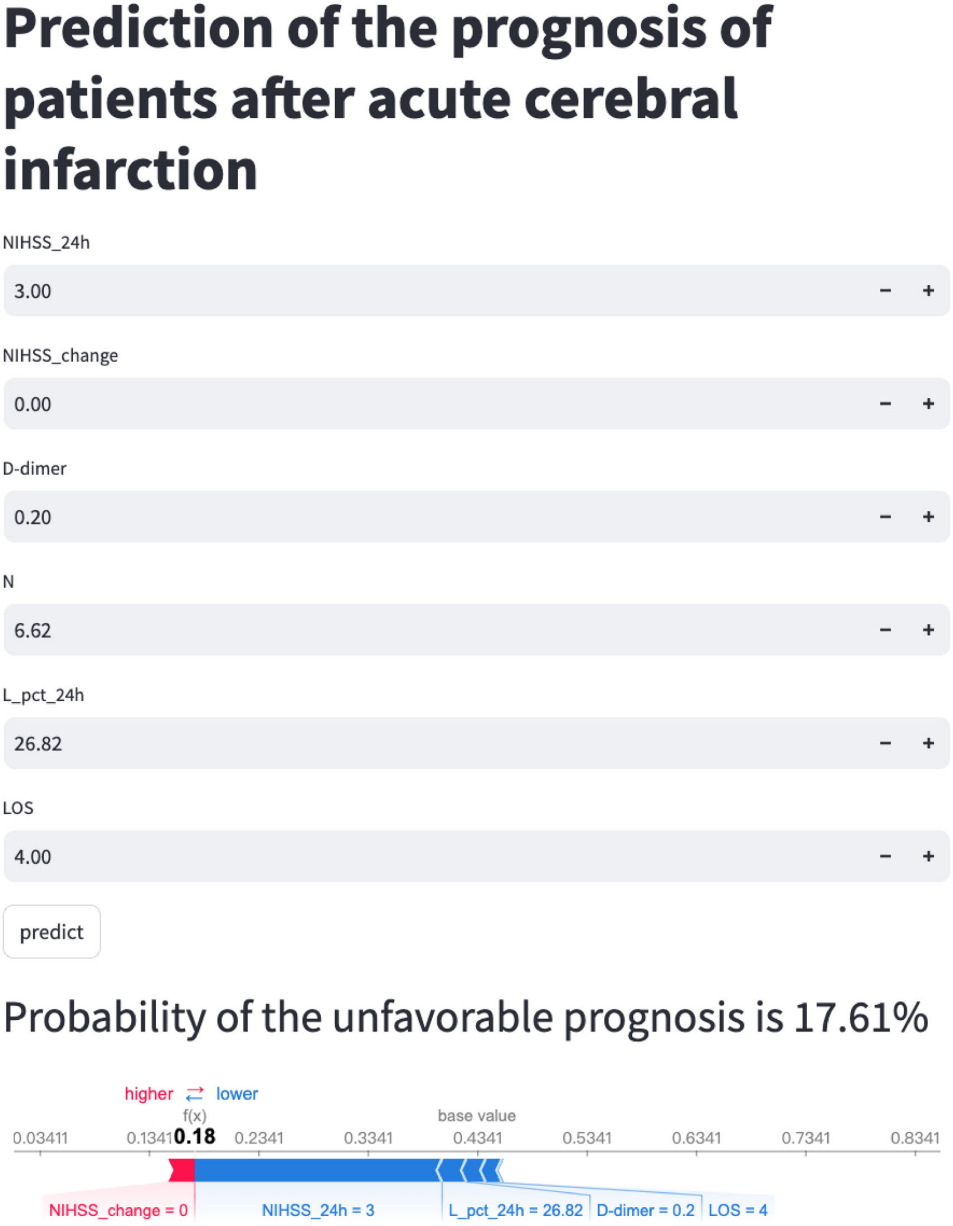
**User‐friendly application for clinical utility**. This web application employs the final LR model with six features for prognosis prediction. Upon entering the actual feature values, the application displays a probability of 17.61%. The force plot for individual patients illustrates which features lead to a favorable prediction: blue features on the right promote a “Favorable” outcome, whereas red features on the left indicate an “Unfavorable” classification.

The final predictive model was integrated into a web application to enhance its applicability in clinical settings, as illustrated in Figure 7. When users input the actual values of the six required features, the application automatically calculates the risk of an unfavorable prognosis for the individual patient. A force plot is also generated, highlighting the features influencing the unfavorable prediction: blue features on the right contribute to a “Favorable” prediction, while red features on the left push towards “Unfavorable”. The web application is accessible at https://predict‐aci.streamlit.app/.

To support clinical implementation, the tool can be integrated into hospital electronic health record (EHR) systems to provide automated, real‐time risk alerts based on early clinical and laboratory data (e.g., NIHSS_24 h, D‐dimer). In practice, a high‐risk prediction could trigger multidisciplinary case discussions, escalation of care (e.g., admission to a stroke unit), or early rehabilitation planning. This integration may enhance decision‐making during the acute phase and improve resource allocation.

## 4 Discussion

This study developed a predictive model for the prognosis of ACI patients using six key indicators: NIHSS_24 h, NIHSS_change, D‐dimer, N, L_pct_24 h, and LOS. Among these, NIHSS_24h and NIHSS change emerged as particularly valuable for early clinical decision‐making. These indicators effectively signaled the likelihood of three‐month post‐discharge outcomes, with higher NIHSS scores within the first 24 h strongly associated with unfavorable prognoses (You et al. 2024). This underscores their utility in guiding initial treatment strategies, essentially providing an early warning system for poor outcomes. Additionally, the inclusion of D‐dimer and L_pct_24 h in the model highlights the importance of coagulation and immune dysregulation in acute ischemic stroke prognosis (Arvin et al. 1996; Liberale et al. 2017). Elevated D‐dimer reflects increased thrombotic burden and fibrinolytic activity, which are associated with larger infarct size and poorer outcomes. Meanwhile, decreased L_pct_24 h indicates alterations in immunosuppression and systemic inflammation, both of which are key determinants of post‐stroke complications and recovery trajectory (Arvin et al. 1996). The integration of these inflammation‐related indicators significantly enhanced the model's predictive power, demonstrating that a multifaceted approach—combining neurological assessments and inflammatory biomarkers—yields superior predictive accuracy. not only improve the predictive accuracy of the model but also provide potential targets for therapeutic intervention. Additionally, the application of SHAP improved the interpretability of predictions by highlighting the contribution of individual features to the model's output. This transparency helps clinicians understand key risk factors influencing patient outcomes, supporting more informed decision‐making in stroke management.

Our model demonstrated higher predictive accuracy (AUC: 0.93 vs. 0.81 and 0.80) and robustness in patients with posterior circulation stroke compared to conventional models such as DRAGON and ASTRAL (Cooray et al. 2016). Unlike traditional prognostic models such as SITS or DRAGON, which rely primarily on static clinical features, our model integrates dynamic biomarkers and advanced machine learning techniques to capture complex interactions. Previous studies have emphasized the respective importance of NIHSS scores and inflammatory indicators. For example, Adams et al. (Tuttolomondo et al. 2012) emphasized the role of early stroke severity assessment using NIHSS, while Di Napoli et al. (Lattanzi et al. 2021) focused on the predictive value of D‐dimer levels in stroke patients. Our study builds on these findings by integrating multiple domains into a single predictive framework, thereby improving both the accuracy and interpretability of the model. In comparison to existing models that often rely solely on clinical or imaging data, our approach provides a more holistic view of the patient's condition. For example, a study by Lees et al. (2010) focused primarily on NIHSS scores and their changes over time but did not incorporate inflammatory markers. By incorporating these additional variables, our model provides a more nuanced and comprehensive understanding of the prognosis of stroke, which is critical for personalizing treatment plans.

The predictive model developed in this study holds significant potential for clinical application. By accurately forecasting patient outcomes, it can assist clinicians in making informed decisions regarding the intensity of early interventions and long‐term management strategies. The inclusion of NIHSS_24h and NIHSS change as key predictors underscores their utility in the early assessment of stroke severity, which can guide immediate therapeutic actions. High NIHSS scores within the first 24 h indicate a need for more aggressive treatment strategies, including prolonged stroke unit monitoring, early neurorehabilitation planning, and timely initiation of secondary prevention measures, per AHA/ASA guidelines. For instance, a patient presenting with NIHSS_24 h = 9 and D‐dimer = 1.5 mg/L was classified as high‐risk by our model, prompting close monitoring for neurological deterioration, early initiation of dual antiplatelet therapy, and a structured rehabilitation plan. Conversely, a patient with NIHSS_24 h = 3 and stable inflammatory markers was considered low‐risk, facilitating early discharge and outpatient follow‐up. Furthermore, the model's reliance on inflammatory markers highlights the importance of monitoring these indicators, which can be targeted for personalized treatment plans. For instance, elevated D‐dimer levels may prompt the use of anticoagulation therapy, while abnormal lymphocyte percentage might suggest the need for immune modulation.

Prognostic models developed using readily available clinical features and biological markers routinely obtained in emergency and inpatient settings have demonstrated robust performance in predicting patient outcomes. These models are practical for clinical use, enabling physicians to make informed treatment decisions and assisting patients in setting realistic recovery goals, thus promoting functional rehabilitation and effective physician‐patient communication. Moreover, the application of streamlined feature selection enhances predictive accuracy and facilitates broader clinical implementation. The integration of web‐based platforms further improves accessibility, allowing for real‐time outcome prediction and decision support in clinical settings, ultimately enhancing resource allocation and patient prognosis. For example, our web‐based tool integrates seamlessly with electronic health record (EHR) systems, providing immediate risk stratification. A high‐risk classification (e.g., NIHSS_24 h ≥ 7, D‐dimer > 1.5 mg/L) triggers an alert recommending intensive monitoring and early rehabilitation referral, whereas low‐risk patients can be prioritized for expedited discharge planning.

One of the primary strengths of this study is its robust dataset, derived from a representative hospital in the eastern Beijing region, Luhe Hospital. Luhe Hospital serves as a cornerstone healthcare facility for the Tongzhou district, catering to a large and diverse population from birth to death. This comprehensive coverage ensures a high degree of internal validity, as the patient cohort closely reflects the broader community's demographics and health characteristics. The hospital's extensive electronic medical records system provided a rich source of data, enabling the development of a highly reliable and generalizable predictive model.

Additionally, the use of machine learning techniques, validated through rigorous internal testing, further strengthens the study's findings. The LR model was selected for its superior performance and interpretability, offering a balance between accuracy and clinical utility. The inclusion of the SHAP method for feature interpretation adds another layer of strength, making the model's predictions more transparent and actionable for clinicians.

Our model enables risk‐stratified clinical decisions by linking key predictors (e.g., NIHSS change > 0) to actionable interventions. High‐risk patients may benefit from early neuroprotective therapies (e.g., dual antiplatelet therapy), intensified monitoring (e.g., repeat neuroimaging), and personalized anticoagulation, while low‐risk cases could prioritize early rehabilitation and resource optimization. The web‐based tool integrates seamlessly with EHR systems, providing real‐time risk alerts alongside SHAP explanations (e.g., feature contribution scores) to guide clinicians during routine assessments. For example, a patient with elevated D‐dimer and NIHSS_24 h would trigger recommendations for statin intensification and extended follow‐up, aligning with guideline‐directed care. Prospective validation in real‐world clinical settings is necessary to further evaluate the impact of model‐based decision‐making on patient outcomes and healthcare resource allocation.

One major limitation of this study is its single‐center design, which may affect external generalizability. Despite its strengths, this study has several limitations. First, the data were collected from a single hospital, which may limit the model's generalizability to other regions or healthcare settings. Although Luhe Hospital is representative of the Tongzhou district, regional variations in healthcare delivery and patient demographics could affect the model's performance elsewhere. Second, although the study included a comprehensive set of predictive features, other potentially relevant variables, such as genetic factors or socioeconomic status, and other composite inflammatory indices, such as NLR and PLR, were not included in this model, as they were not precomputed and were beyond the scope of our initial variable selection strategy focused on early, raw clinical markers. Future studies may examine the added value of these derived metrics. These factors could influence stroke outcomes and should be included in future models.

Future research should aim to validate the predictive model in multi‐center studies to enhance its generalizability. Incorporating additional variables, such as genetic data and lifestyle factors, could further improve the model's accuracy and applicability.

Additionally, exploring the potential for integrating this model with other predictive tools and decision support systems could yield comprehensive solutions for ACI management. For example, combining our model with imaging data or genetic information could provide a more complete picture of stroke risk and prognosis. Collaborations with other institutions and healthcare systems will be crucial for these efforts, ensuring that the model can be adapted to diverse clinical environments.

## 5 Conclusion

In conclusion, this study presents a robust and interpretable predictive model for the prognosis of acute cerebral infarction patients, utilizing nine key indicators. The model's integration of NIHSS_24 h, NIHSS change, and inflammatory markers provides valuable insights into patient outcomes, facilitating early clinical decision‐making. The study's strengths, including its comprehensive dataset and rigorous validation, underscore the model's potential for widespread application. However, further validation and refinement are necessary to address the study's limitations and to enhance its clinical utility. Future directions include multi‐center validation and the incorporation of additional predictive variables to improve the model's accuracy and generalizability.

## Author Contributions

**Linlin Ma**: writing–original draft, data curation, methodology. **Lang Ji**: software, methodology, formal analysis, visualization. **Zhe Cheng**: project administration, validation, investigation. **Xiaokun Geng**: supervision, writing–review and editing, funding acquisition, formal analysis, project administration, resources. **Yuchuan Ding**: supervision, project administration, writing–review and editing, resources, conceptualization, funding acquisition.

## Ethics Statement

This study was a real‐world, retrospective cohort analysis using de‐identified patient data. Informed consent was not required as per the ethical guidelines for retrospective studies. The study was approved by the Institutional Review Board (IRB) of Beijing Luhe Hospital. All procedures followed the ethical principles of the Declaration of Helsinki.

## Conflicts of Interest

The authors declare no conflicts of interest.

## Peer Review

The peer review history for this article is available at https://publons.com/publon/10.1002/brb3.70673

## Supporting information




**Supplementary Material**: Appendix brb370673‐sup‐0001‐Appendix.docx

## Data Availability

The data that support the findings of this study are available on request from the corresponding author. The data are not publicly available due to privacy or ethical restrictions.
