# Supplementary material for: Developing an Explainable Prognostic Model for Acute Ischemic Stroke: Combining Clinical and Inflammatory Biomarkers With Machine Learning
Source: Brain Behav. 2025 Jul 31;15(8):e70673. doi: 10.1002/brb3.70673 (PMC12311615; doi:10.1002/brb3.70673)
Supplement: Supplementary file 1 — Supplementary Material: Appendix brb370673‐sup‐0001‐Appendix.docx [file BRB3-15-e70673-s001.docx]

****Appendix S1:** ROC Curves for All 12 Models**


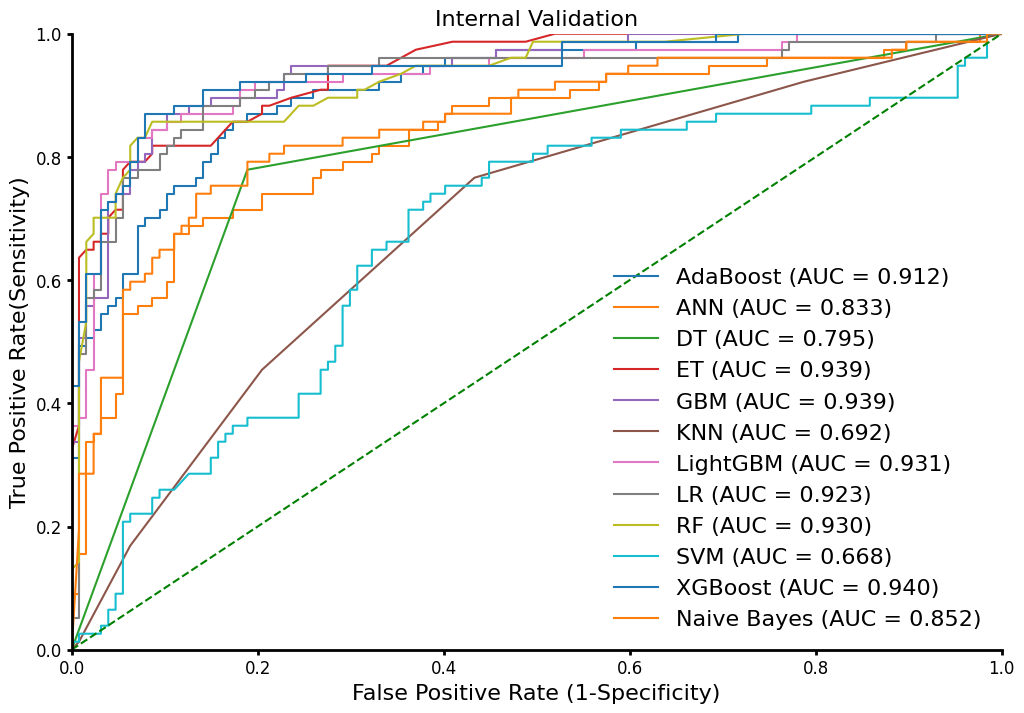


****Appendix S2: AUC Values for the Top 7 Models Using 1 to 28 Features****


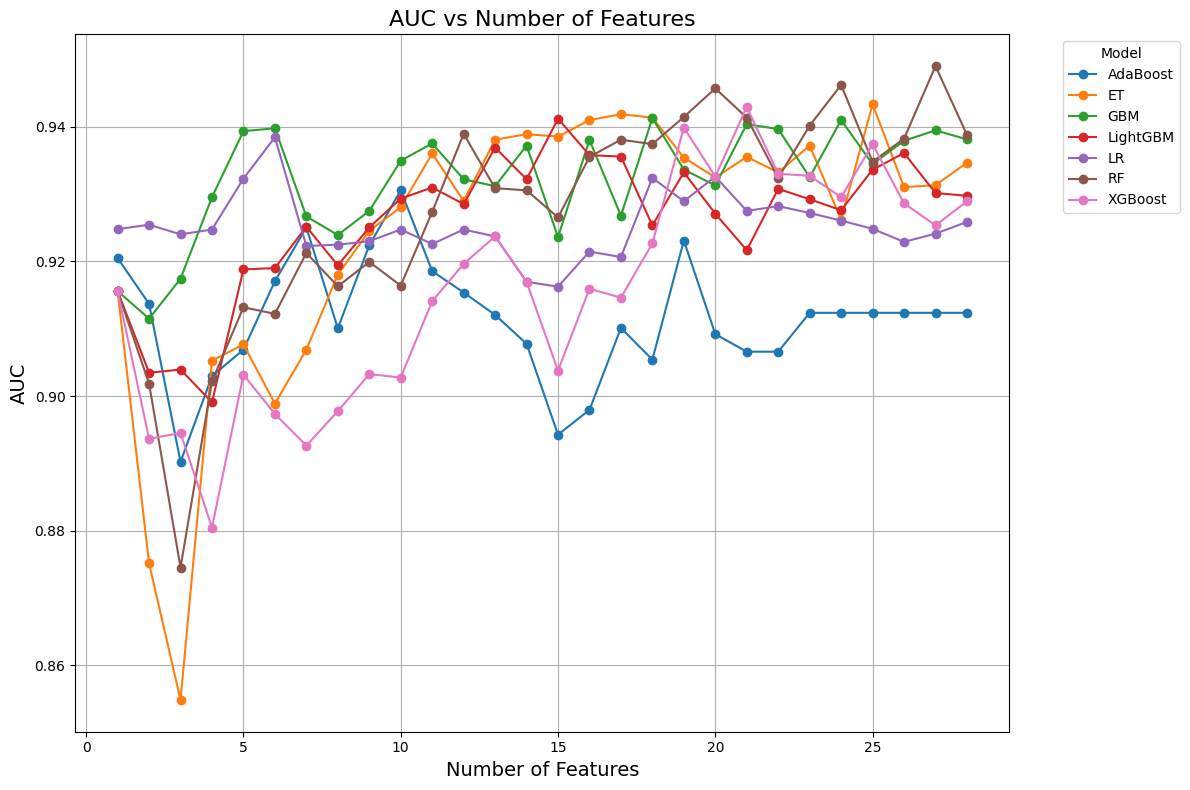


| ****Appendix S3:**Ten-Fold Cross-Validation Results for the Top 7 Models** | | | |
| --- | --- | --- | --- |
| Model | Mean_AUC | Std_AUC | All_Scores |
| AdaBoost | 0.8169651068941569 | 0.16942074094070045 | 0.50965708 0.93141506 0.96886086 0.92116673 0.95348837 0.96649586 0.9625542 0.62227603 0.68119451 0.65254237 |
| ET | 0.9346589522683335 | 0.10289174439842569 | 0.97122586  1. 0.99960583 0.99822625 0.9988175 0.997635 0.99467875 0.91686844 0.74132365 0.72820823 |
| GBM | 0.8404714980197834 | 0.18423369369472656 | 0.60465116 0.93929838  1. 0.95743004 0.98541585 0.99605834 0.99369334 0.7905569 0.47901533 0.65859564 |
| LightGBM | 0.8342849634926891 | 0.20250346982158693 | 0.63106031 0.94087505 0.99960583 0.99093417 0.99290501 0.98068585 0.97950335 0.75464084 0.37207425 0.70056497 |
| LR | 0.9546314544737878 | 0.07767461344001009 | 0.99211667  1.  1.  1.  1. 0.99960583 0.97832085 0.95843422 0.86198547 0.75585149 |
| RF | 0.886857837340691 | 0.14899720493784138 | 0.84410721 0.96472211        1. 0.99901458 0.82828894 0.63054883 0.60189669] |
| XGBoost | 0.8362342098841902 | 0.1837486421766944 | 0.67067402 0.90973591 0.9940875 0.98423335 0.99172251 0.97635002 0.97516752 0.7905569 0.46004843 0.60976594 |

****Appendix S4:** SHAP Summary Plot for All 28 Features in the Logistic Regression Model**


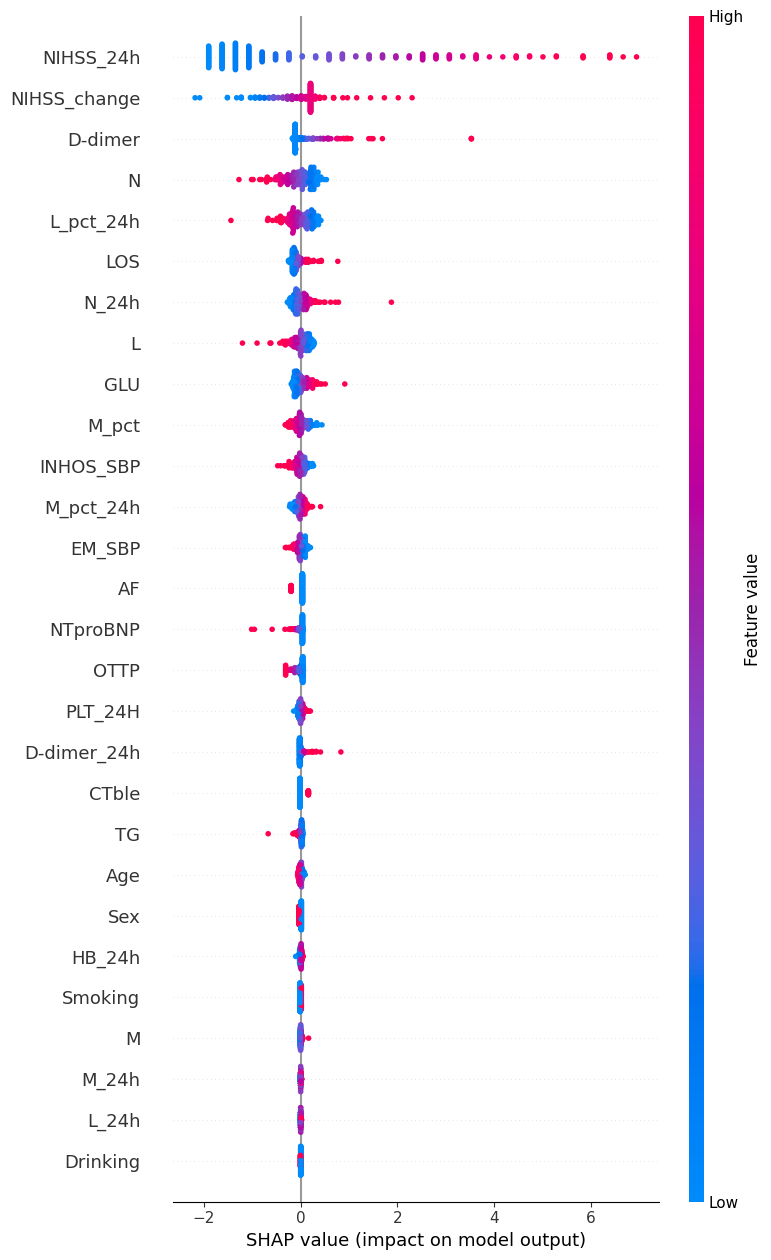


| ****Appendix S5:**** Detailed metrics for the seven models | | | | |
| --- | --- | --- | --- | --- |
| Model | Accuracy | AUC | Sensitivity | Specificity |
| AdaBoost | 0.848039 | 0.901998 | 0.788889 | 0.894737 |
| ET | 0.852941 | 0.919737 | 0.722222 | 0.956140 |
| GBM | 0.833333 | 0.929825 | 0.688889 | 0.947368 |
| LightGBM | 0.862745 | 0.930799 | 0.733333 | 0.964912 |
| LR | 0.872549 | 0.934113 | 0.766667 | 0.956140 |
| RF | 0.838235 | 0.916082 | 0.700000 | 0.947368 |
| XGBoost | 0.838235 | 0.919786 | 0.711111 | 0.938596 |
